# The need for patient-centred clinical research in idiopathic pulmonary fibrosis

**DOI:** 10.1186/s12916-015-0475-4

**Published:** 2015-09-24

**Authors:** Anne-Marie Russell, Mirjam AG Sprangers, Steven Wibberley, Noel Snell, Daniel M. Rose, Jeff J. Swigris

**Affiliations:** NIHR Clinical Research Fellow Imperial College National Heart & Lung Institute and Royal Brompton Hospital, Emmanuel Kaye Building, Manresa Road, London, SW3 6LR UK; Department of Medical Psychology, Academic Medical Centre, University of Amsterdam, Meibergdreef 15, 1105 AZ Amsterdam, The Netherlands; British Lung Foundation, 73-75 Goswell Road, London, EC1V 7ER UK; Pulmonary Fibrosis Foundation, 230 East Ohio Street, Suite 304, Chicago, IL 60611 USA; Interstitial Lung Disease Program, National Jewish Health, Southside Building, Office G011 1400 Jackson Street, Denver, CO 80206 USA

**Keywords:** Clinical research, Decision-making, Idiopathic pulmonary fibrosis, Patient-centredness, Patient-reported outcomes, Patient co-investigator

## Abstract

Patient-centredness is an accepted term and is perceived by healthcare professionals to be morally and ethically desirable. We are motivated by the belief that this approach will improve the patient-professional experience of the decision-making process and improve health outcomes. We acknowledge that patients, either as participants or as co-investigators, have positive contributions to make to research. As the idiopathic pulmonary fibrosis (IPF) community enters a new era of clinical research activity we consider that there is greater capacity for patient involvement and partnership.

Patient involvement in research can be optimised through collaborations in the research design, study conduct, and dissemination. There is increasing interest in using patient- reported outcomes (PROs), such as health-related quality of life, and symptoms measures to inform decision-making and ensure patient perspectives are taken into account. PROs are an essential component of specialist IPF services, to monitor and improve care delivery and to measure and benchmark performance. In clinical trials, PROs can additionally be used to define entry criteria, evaluate efficacy of an intervention, and evaluate adverse events. We suggest that there is a much wider scope for including patient-centred PROs in clinical research and for creative thought in developing patient co-investigator roles.

Participation in research activity requires highly refined decision-making processes, particularly in a condition such as IPF, which has an often unpredictable trajectory. The IPF research landscape has changed and the design and conduct of clinical trials in IPF requires some radical rethinking. It is accepted that involving patients in the role of co-investigators will impact the research questions we ask and result in study designs that are patient-centred. IPF clinical trials have been hindered by the lack of availability of validated, disease-specific questionnaires. A conservative approach appears to have been taken to the inclusion of generic symptom or quality of life measures as PRO endpoints. Thus, the impact of new drugs on the quality of life of research participants demonstrates only minimal benefit. It is time to refocus on a patient-centred approach with regards to the co-investigator role, PRO development, and research participants.

## Background

Patient-centredness, as a broad construct, has been discussed in the literature for more than three decades. It experienced resurgence in the “Patients Included” initiative [1], interfacing with patient and public involvement in research. From the policy perspective, the Institute of Medicine defines patient-centred care as a priority domain of high quality healthcare:

“*a partnership among practitioners, patients, and their families (when appropriate) to ensure that decisions respect patients’ wants, needs, and preferences and that patients have the education and support they need to make decisions and participate in their own care*” ([2], p. 169).

Patients diagnosed with idiopathic pulmonary fibrosis (IPF) and their families have expressed a need for more healthcare support and education [3, 4], including prompt referral to specialist centres and timely diagnosis [5]. Whilst we can learn from the shared generic symptom experience and standardised approaches in cancer and chronic diseases, IPF can have inherent diagnostic and management challenges. A chronic, progressive, fibrotic lung disease, IPF follows an unpredictable course; typically affecting older people (who may have pre-existing co-morbidities) and has a 5-year survival of only 20 % [6]. The symptoms associated with IPF include breathlessness, cough, fatigue, anxiety, and depression [3, 4]. Patients also report concerns about loss of fitness, loss of weight, and the impact of both the condition and interventions on their nutritional state [7]. Therefore, involving patients in the decision-making process on all aspects of care management and participation in research requires some expediency.

Recent priorities in IPF have focussed on understanding the natural history of the disease and the pathobiology. Progress to date has enhanced this understanding [8] and delivered two new effective pharmacological therapies that appear to slow disease progression: pirfenidone (Esbriet: InterMune®), approved for use in Europe (in 2011), the UK (in 2013), and the US (in 2014) and nintedanib (OFEV: Boerhinger®), awaiting final approvals in the UK and approved by the US Federal Drug Administration (FDA) in 2014. Nevertheless, there is still a need to characterise the impacts of these therapies on patients’ perceived health status and overall quality of life. The next generation of IPF clinical trials will require design modifications to account for the availability of existing therapies [9]. Collard et al. [9] propose a coalition of stakeholders including patients and advocacy groups to contribute to trial design bringing creativity and flexibility. As a rare disease, IPF research will require global collaboration to combine efforts, standardise approaches, and share expertise. Patient organisations are particularly important, offering additional incentives.

IPF researchers have experienced challenges due to the lack of consensus in optimal outcome measures in IPF trials [10–14]. Although some experts believe that it is not a surrogate for mortality [15], forced vital capacity (FVC) has become the favoured primary endpoint amongst trial designers. This is due, in part, to its reliability and reproducibility across pulmonary function testing laboratories and equipment and its ability to predict mortality in several observational studies.

Meanwhile, patient-centred outcomes, such as self-report measures to assess how patients feel and function in their daily lives, have been, at best, included as lower-tier endpoints. Relegation of patient-centred outcomes likely stems from an absence of IPF-specific measures, a lack of longitudinal data to support validity, and uncertainty regarding the significance of score values or minimal important differences. This impacts the meaningfulness of many data generated in IPF trials, shifting the focus away from the patient experience.

We support the view of Collard et al. [9] calling for a shift in emphasis in IPF clinical research and trial design. We propose a strategy that places the patient at the heart of research to enrich our understanding of what living with IPF is like. Primarily, we advocate a pragmatic approach in selecting methodologies best suited to the research problem; mixed methods that employ both quantitative and qualitative designs should be considered, acknowledging the limitations and complementary nature of both, followed by the involvement of patients in the research process as active investigators, and finally, paying closer attention to the choice of patient-centred outcomes to ensure we are measuring what is important to patients. The selection of patient-reported outcomes (PROs) based on familiarity is no longer acceptable.

## Discussion

### Patient-centred research

The Patient-Centred Outcomes Research Institute (PCORI) strives to generate reliable data that patients, caregivers, and practitioners can use to make informed decisions and to assess the value of healthcare options [16]. An evaluation protocol is proposed by the Patient-Centred Outcomes Research trust fund to determine how patient-centred research impacts healthcare [17]. Studies funded by PCORI will be evaluated according to three components: financial accountability, level of adherence to the principles of patient-centredness (defined as relevant, pragmatic, feasible, and participatory), and whether they make a difference to healthcare quality outcomes.

In the UK, the National Institute for Health Research has funded, for two decades, a national advisory group, INVOLVE^©^, to promote and monitor active public involvement in National Health Service (NHS) public health and social care research. Many research funding streams in the US, Netherlands, and the UK (including National Institute for Health Research funded studies) require well-documented evidence of patient/public involvement from protocol development through to dissemination and implementation of results. Ethical review boards also ensure patient’s interests are represented at the outset.

Recently formed working groups with a remit to determine future directions in IPF research [18] or to identify core sets of domains and instruments for use in clinical trials [19] have included patient representatives; unfortunately, there is no mention of any patient input to a recent UK round table discussion on improving care for IPF patients [20]. The British Lung Foundation has identified IPF as a research priority and includes patient representatives on its IPF research advisory board. International IPF-specific charities and advocacy groups have developed a strong patient voice discussing and disseminating research information. The International Society for Quality of Life (ISOQOL) hosts a Special Interest Group for patient and carer engagement in health related quality of life (HRQoL) research.

The American Thoracic Society/European Respiratory Society/Japanese Respiratory Society/Latin American Thoracic Association Clinical Practice Guideline [21] development group included a patient representative and his positive contribution was extensively acknowledged. Sadly, he passed away before the guideline was published, highlighting the dilemma faced by IPF researchers/clinicians. IPF is associated with an unpredictable trajectory and high mortality. Research processes from conception, through design, ethical approvals, study conduct, and dissemination are lengthy. Realistic planning and sensitive thought needs to be given as to how to engage with patients in the most meaningful way. It may be expedient to collaborate with larger patient/carer groups, under agreed terms of reference with clearly defined roles.

Essential components of patient-centred approaches include understanding what is important to patients, respecting each individual’s health beliefs, values, and judgments [22]. Measuring levels of patient satisfaction correlates with participation in research [16], although it is likely that satisfaction is a more sensitive marker of the patient-professional relationship than the outcome *per se*. Nonetheless, ‘satisfaction’ should be considered as an outcome measure in the research encounter.

### Drivers for PROs

In the UK, there has been a recurring call for patient involvement in health service development and delivery (progressively including research) since 2002 [23–26]. However, the UK is divided. England places greater emphasis than its devolved nations on patient choice and transparency in the NHS and, in what is perceived as a landmark development, introduced a PROs programme to measure patients’ self-reported health status in 2009. Initially, four surgical procedures (inguinal hernia repair, hip and knee replacements, and varicose vein surgery) were included. The NHS Executive has subsequently extended the PRO programme to cancer, mental ill-health, and six chronic conditions, including asthma and chronic obstructive pulmonary disease [27].

The National Institute for Health and Care Excellence (NICE) Quality Standards have a wider scope to drive the use of PROs, generating patient-derived outcome data and measuring change in HRQoL of both patients and carers as benchmarks that commissioners and providers use as performance indicators for NHS care. The 24 designated specialist centres for Interstitial Lung Diseases (and IPF in particular) in the UK must adhere to NICE Quality Standards [28]. This core minimum standard is enhanced by the NICE Clinical guideline [29] that advocates patient-centred care in the diagnosis and management of IPF. PCORI, in the US, advocates the use of PROs with equal enthusiasm [30].

### Patient-centred outcomes

The most well-known patient-centred outcomes are PRO measures that capture what is meaningful and valuable to the individual patient [31]. Confirming what is meaningful is accomplished through open dialogue with patients and must be initiated at the beginning of the process. In fact, the entire process of endpoint development and selection must be built around – and ideally in part, by – the patient, rather than the clinician-investigator. A PRO is defined as:

“*Any report coming directly from the patient without interpretation by a third party about how they feel or function in relation to a health condition and a given intervention*” [32].

Collection of PRO data enables clinicians to access information on outcome variations, to advance person-centredness, and to capture the physical, emotional, and social domains of health that will be important for healthcare provision. This has been shown to impact clinician-patient communication [33–35], assist with shared decision-making [36], and contribute to the success of the encounter [37].

A PRO must also meet psychometric criteria that any other useful clinical endpoint is required to meet. It should be reliable (scores remain unchanged if there is no change in the construct it measures), valid (measure what it purports to measure), and responsive to changes in the construct it measures (regardless of the aetiology of those changes) [38]. It is helpful if scores are predictive of clinical outcome and a threshold for minimal important differences is known [39].

The frequency with which PRO data are collected is variable. ISOQOL recommend that condition-specific PRO data be collected at least every 6 months; for patients requiring regular treatment, such as chemotherapy, weekly data collection is recommended [40]. In clinical research, a PRO can facilitate discussion, promote self-efficacy, improve outcomes, increase satisfaction, and improve adherence to and measurement of the impact of the intervention. Given the uncertain trajectory of IPF, we advocate PROs are administered at all points of significant transition – for example, at exacerbation or at dose titration of therapies such as pirfenidone and nintedanib. The IPF integrated care pathway (a tool that formalises the care processes) must be able to accommodate this.

### Selecting a PRO for IPF

The main endpoints used in IPF research have typically included time to disease progression, transplant, or death; disease progression (defined by decline in FVC, diffusing capacity of the lung for carbon monoxide, or distance walked in 6-minute walk test); or the occurrence of complications (respiratory failure, hospitalization, or acute exacerbations). Whilst these clinical endpoints offer objective measures of decline, they fail to directly capture aspects most meaningful to patients, such as symptom frequency/severity, physical functional status, level of independence, social functioning, and psychological state.

NICE recommends that interventional studies of patients with IPF should include, as endpoints, a breathlessness score and a measure of HRQoL that have been validated in an IPF cohort [29]. The European Lung Foundation emphasizes the importance of enabling patients with IPF to be able to report on their quality of life, in particular to facilitate discussion on the IPF-imposed need to adjust life goals [7].

Symptoms measures, health status, and quality of life questionnaires are being integrated into IPF research, but there is a limited number of candidate PROs validated for use in IPF cohorts. Given the new era of clinical trials, we need to be able to select from a range of complementary patient-centred PROs that conform to FDA criteria, have robust psychometric properties, and are available in multiple languages to enable global collaboration. IPF Registries may offer the opportunity to create larger data sets to enable longitudinal evaluation of IPF PROs.

Currently, there is only one IPF-specific PRO: A Tool to Assess Quality of Life in IPF (ATAQ-I) [41]. It was created using methodology concordant with FDA recommendations (i.e. originating through open-floor, patient-led discussion) [31]. The original version with 89 items is associated with a moderate burden to complete. The ATAQ-I has been modified [42] and a subsequent modified version has been submitted for FDA approval. Prospective studies are still needed to establish the ATAQ-I specificity in IPF populations.

The St George’s Respiratory Questionnaire (SGRQ) [43] has been used in IPF studies and its psychometric properties reviewed in 30 studies to date [44]. The impact and activity domains and the total score were found to have greater reliability to detect change in health status compared with the symptom domain in IPF populations. This was most evident in the INPULSIS I and INPULSIS II phase 3 trials to investigate the impact of nintedanib on IPF [45]. Whilst the SGRQ is not the optimal PRO for HRQoL, it is considered acceptable for use in IPF populations. An IPF-specific modified version has also been developed: the SGRQ-I (SGRQ–IPF) [46].

Another HRQoL measure for patients diagnosed with interstitial lung disease has also been developed in a population with predominantly connective tissue interstitial lung disease, the King’s Brief Interstitial Lung Disease questionnaire [47]. Its utility in IPF is currently been explored. We await reports from longitudinal studies on its stability as well as that of the SGRQ-I and ATAQ-I in IPF populations over time.

A body of literature exists to support the prognostic value of the generic Medical Research Council (MRC) dyspnoea scale as an outcome measure in IPF. Patient-reported dyspnoea has been shown to be the single strongest predictor of mortality in patients with IPF [48] and correlates strongly with quality of life [49]. The modified MRC score has been shown to have prognostic value in predicting survival [50] and in detecting disease progression [51] in IPF. Holland et al. [52] reported that the MRC score is also an independent predictor of anxiety and depression in a mixed interstitial lung disease cohort. As a five-item questionnaire, the burden is light and the MRC is suitable for both clinical and research contexts.

The University of California San Diego Shortness of Breath Questionnaire [53], frequently cited in the IPF literature, was used as a secondary outcome measure in the Capacity (phase II and III) [54–56] and Ascend (phase III) trials [57]. Whilst the rate of decline of FVC in the treatment group was significantly reduced (*P* <0.001) and evident by week 13, the impact of pirfenidone on the experience of breathlessness did not achieve statistical significance with the questionnaire in this study.

The PROs used to date in IPF research fail to address certain aspects deemed important by patients. For example, fatigue is a greater concern than breathlessness for many patients with IPF [58]. Further, whilst textbooks describe dry cough in patients with IPF, patients often report being troubled by productive cough and describe exhausting regimens of phlegm clearance. Without engaging with patients such important aspects are missed.

### Patients as co-investigators

We believe that conducting qualitative research (i.e. in-depth patient interviews and/or focus groups) prior to designing a study is paramount to patient-centred research, including patients as members of the research team and using their expertise – they are, after all, the true experts on living with their disease – to define and refine interventions, select outcome measures, and disseminate results [59–62]. We increasingly hear the patient voice at international respiratory conferences, where a unique perspective is offered complementing that of the clinician-researcher.

Contributing to basic science and pre-clinical research may be more challenging and requires a greater level of technical knowledge and specific training for the research team. However, it is proposed that patient involvement in non-clinical research gives rise to experiential knowledge that adds validity to the process. The challenge lies in translating and applying this knowledge appropriately [63].

Patients’ expertise lies in conveying what it means to live with IPF and how living with IPF impacts life in all its domains. Given the inherent morbidity and mortality associated with IPF, a flexible approach is needed as to how to operationalise the inclusion of patients on the team. Patients are not always able to travel. Telephone conference facilities should be available, supplemental oxygen provided to patients who need it during meetings, meetings should be relatively short and planned well in advance, and training germane to the research must be identified and offered. Thought needs to be given to appropriate remuneration if patients are to be truly perceived as partners/co-investigators.

Patient-centredness may also include the involvement of care-givers as co-investigators. Caregivers are invaluable enablers in the research process offering further insight into the perspective of the one they care for.

## Summary

A more patient-centred approach in this new era of IPF research is an urgent priority. Expanding patient consultation in research design processes, to ensure that research questions address what is important to patient populations, and subsequently actively involving patients in the conduct of studies, the dissemination process, and ongoing collaboration is achievable. Patients or their designated carer must also receive feedback regarding the results of the research in which they have participated. This is likely to assist with retention and future recruitment. It is essential that we capture patients’ level of satisfaction with all aspects of the research process.

As new therapies for IPF emerge, individualised approaches may result in new regimens of combination therapies. Collecting PROs has never been more important to enable us to determine what is acceptable and tolerable for an individual over time. In addition, these data collectively determine the impact of ‘supportive care only*’* and ‘single’ or ‘combined therapies’ on health status in IPF populations. We must critically appraise our choice of PRO and the time points at which it is administered.

Patient centred research is a dynamic process achieved by involving and working with patients at all levels from pre-clinical research and clinical trials through to post-marketing surveillance. Contemporary patient-centred PROs are not without limitation but they do add value and remind and enable us to regard the patient before the condition.

## Abbreviations

ATAQ-I: A Tool to Assess Quality of Life in IPF
FDA: Federal Drug Administration
FVC: Forced vital capacity
HRQoL: Health related quality of life
ISOQOL: International Society for Quality of Life Research
MRC: Medical Research Council
NICE: National Institute for Health and Care Excellence
NHS: National Health Service
PCORI: Patient-Centred Outcomes Research Institute
PRO: Patient-reported outcomes
SGRQ: St George’s Respiratory Questionnaire
